# Supplementary material for: Habitat use by female desert tortoises suggests tradeoffs between resource use and risk avoidance
Source: PLoS One. 2022 Aug 19;17(8):e0263743. doi: 10.1371/journal.pone.0263743 (PMC9390940; doi:10.1371/journal.pone.0263743)
Supplement: S1 Table — This is a complete list of all species that were recorded to be present during the sampling of habitat around Mojave desert tortoise occupied burrows and a paired point within 200 m of the burrow. (DOCX) [file pone.0263743.s001.docx]

Supplemental Table 1: Ephemeral forb and grass species documented in 1 m x 1m during summer 2012 (August 15 – 30)

| Ephemeral Forbs and Grass | | |
| --- | --- | --- |
|  |  |  |
| **Forbs** |  | **Grasses** |
| *Allionia incarnata* |  | *Bouteloua aristidoides* |
| *Amaranthus fimbriatus* |  | *B. barbata* |
| *Boerhavia triquetra* |  | *Oryzopsis hymenoides* |
| *Calyptridium monandrum* |  | *Schismus barbarata* |
| *Chamaesyce polycarpa* |  |  |
| *C. setiloba* |  |  |
| *Eriogonum deflexum* |  |  |
| *Kallstroemia californica* |  |  |
| *Pectis papposa* |  |  |
| *Portulaca halimoides* |  |  |
| *Tribulus terrestris* |  |  |
